# What do health care professionals want to know about assisted dying? Setting the research agenda in New Zealand

**DOI:** 10.1186/s12904-023-01159-8

**Published:** 2023-04-10

**Authors:** Jessica Young, Jeanne Snelling, Sophie Beaumont, Kate Diesfeld, Ben White, Lindy Willmott, Jacqualine Robinson, Annabel Ahuriri-Driscoll, Gary Cheung, Aida Dehkhoda, Richard Egan, James Jap, Te Hurinui Karaka-Clarke, Leanne Manson, Cam McLaren, Janine Winters

**Affiliations:** 1 Te Herenga Waka – Victoria University of Wellington, Wellington, New Zealand; 2 University of Otago, Dunedin, New Zealand; 3 Auckland University of Technology, Auckland, New Zealand; 4 Queensland University of Technology, Brisbane, Australia; 5 University of Auckland, Auckland, New Zealand; 6 University of Canterbury, Christchurch, New Zealand; 7 Tōtara Hospice, Auckland, New Zealand; 8 Wellington, New Zealand; 9 Monash University, Melbourne, Australia

**Keywords:** Assisted dying, Research prioritisation, Early implementation, Health professionals

## Abstract

**Background:**

New Zealand recently introduced law permitting terminally ill people to request and receive assisted dying (AD) in specified circumstances. Given the nature and complexity of this new health service, research is vital to determine how AD is operating in practice.

**Objective:**

To identify research priorities regarding the implementation and delivery of AD in New Zealand.

**Methods:**

Using an adapted research prioritisation methodology, the researchers identified 15 potential AD research topics. A mixed-methods survey of health professionals was undertaken where respondents were asked to rate the 15 topics according to the relative importance for research to be conducted on each issue. Respondents could also suggest additional research areas, and were invited to participate in a follow-up interview.

**Results:**

One hundred and nineteen respondents completed the survey. 31% had some experience with AD. The highest rated research topic was the ‘effectiveness of safeguards in the Act to protect people’; the lowest rated topic was research into the ‘experiences of non-provider (e.g., administrative, cleaning) staff where assisted dying is being provided’. Respondents suggested 49 other research topics. Twenty-six interviews were conducted. Thematic analysis of interview data and open-ended survey questions was undertaken. Six research themes were identified: general factors related to the wider health system; the experiences of health care providers at the bedside; medico-legal issues; the impact of AD; experiences on the day of dying; and the overall effectiveness of the AD system. Key issues for stakeholders included safety of the AD service; ensuring access to AD; achieving equity for ‘structurally disadvantaged’ groups; and ensuring the well-being of patients, families/whānau, providers and non-providers.

**Conclusions:**

Based on early experiences of the implementation of the AD service, health professionals provide important insights into what research should be prioritised post-legalisation of AD. These findings can be used to shape the research agenda so that research may inform law, policy and best practice.

**Supplementary Information:**

The online version contains supplementary material available at 10.1186/s12904-023-01159-8.

## Introduction

A growing number of countries have recently introduced laws permitting assisted dying (AD) in specified circumstances (variously called voluntary euthanasia or physician-assisted dying elsewhere). Aotearoa New Zealand (NZ) is one of the latest jurisdictions to enact such legislation [1]. In November 2021, the End of Life Choice Act 2019 (the Act) came into force after a public referendum supported its introduction [2]. Given the nature and complexity of this new health service, in particular concerns regarding safety and accessibility in a bicultural environment unique to Aotearoa New Zealand, [3] research is vital to determine how the service is being implemented and integrated into end of life care [2, 4, 5]. In addition, the Act requires that the operation of the new law be reviewed in 2024 to determine if any amendments to the legislation are “necessary or desirable” [6]. Consequently, there is a pressing need for timely, high-quality AD research to inform the review.

Whilst research on AD was conducted in NZ prior to the recent law reform, [4, 7–19] there is currently no peer-reviewed research about how the AD service operates since the law came into effect. In this article, we describe the first study conducted since AD was introduced in NZ. The aim of this study is to identify research priorities from the perspectives of stakeholders with early experience of AD to inform future research and development of the service.

Engaging stakeholders is a vital part of health research as they can offer invaluable insights that elevate the conduct and impact of research in practice, particularly where there is a lack of research informed evidence [20–22]. A stakeholder is an “individual or group who is responsible for or affected by health- and healthcare-related decisions that can be informed by research evidence” [23]. Undertaking research prioritisation exercises with stakeholders is a well-tested framework for engagement [20–22].

The limited international work on AD research priorities primarily comes from the United Kingdom (UK) where AD is currently being debated, and Belgium where the euthanasia law has been in place since 2002 [24, 25]. A further Canadian study, where AD has been available since 2016, outlines a protocol for exploring key stakeholders’ attitudes and opinions on medical assistance in dying and palliative care [26]. Overall, these studies suggest that stakeholders can identify a range of research areas and priorities [24–26].

To the best of our knowledge, this is the only study using stakeholders to identify research priorities in the early stage of implementing and delivering AD in a jurisdiction post-legalisation. Conducting this type of engagement promises a range of valuable outcomes. It may: identify emergent issues in the implementation; it may give healthcare professionals (HCPs) a platform to provide feedback to inform law, policy and practice; it may provide evidence to dispel or confirm concerns regarding AD; and it may facilitate productive conversations amongst healthcare practitioners who hold differing views on AD.

A further rationale for conducting priority setting research in NZ is because of its unique bi-cultural composition and the Crown’s legislative obligations to the indigenous Māori people. This legal obligation is underpinned by the signing of the Treaty of Waitangi / Te Tiriti o Waitangi in 1840. Internationally, research is required that takes into account the needs of groups whose world view may not align with Western values of death and dying. This has implications for preferences at the end of life including AD. For NZ, meeting Te Tiriti obligations requires that the following principles are considered at every stage of research: tino rangatiratanga (Māori self-determination), ōritetanga (equity), active protection, options, and partnership between Māori and the Crown [27]. Hence while this paper draws on international research prioritisation studies, [24–26] it is necessarily contextualised to NZ’s unique bi-cultural environment.

After briefly outlining the legal criteria for accessing AD in NZ, this paper provides a description of the methodology used. The quantitative and qualitative findings regarding AD research are then presented and synthesised in the discussion. We conclude with a call for further research that aligns with the identified research priorities.

### The End of Life Choice Act (2019)

The Act establishes a highly regulated process, establishes specific eligibility criteria for persons seeking AD, and imposes a range of safeguards that creates new legal obligations for health care providers. A person will only be eligible for AD if at least two medical practitioners, one of whom is appointed by the Ministry of Health, agree: they are aged 18 or over; are a NZ citizen or a permanent resident; they suffer from a “terminal illness likely to end their life within 6 months”; and they are “in an advanced state of irreversible decline in physical capacity”; and they are experiencing “unbearable suffering that cannot be relieved in a manner that the person considers tolerable”; and they are competent to make an informed decision about AD. See supplementary file (Fig. 1) for a simplified overview of the AD process and safeguards. Key differences to other jurisdictions include the requirements of choosing a date (which can be subsequently changed), time and method of medication delivery in advance and obtaining a final notification (in compliance with the Act) from the Registrar before any medication can be dispensed.

## Methods

We used a mixed methods design of an online survey supplemented by individual interviews and a focus group. We focused on two substantive areas of inquiry: questions regarding research prioritisation and early experiences with AD. In this article we report on findings related to research prioritisation. Ethical approval was granted by the Te Herenga Waka – Victoria University Human Ethics Committee #0000030155, #0000030250. All stakeholders provided informed consent prior to participation. To indicate the different participant cohorts, we will refer to people who completed the survey as respondents, interviewees as participants, and collectively as stakeholders.

### Online survey

#### Survey development

The process of developing the survey for this research first involved a review of the research prioritisation literature [28–32] by JY and SB. This was followed by generation of research topics at an online hui (meeting) with a newly formed interdisciplinary AD research network. After the hui the survey was constructed and piloted before being finalised for distribution. See survey supplementary file 2.

#### Literature review

The prioritisation literature indicates that the initial process of research priority setting is traditionally completed with a group of stakeholders, [28] although some studies have used literature reviews [33]. At the time of the current study a COVID-19 outbreak in New Zealand was placing additional pressures on the health care system, making it difficult to access stakeholders. This study adapted the process for the initial identification of research priorities; instead of requiring stakeholders to come up with their own research areas *ab initio*, a list of topics were identified from the AD research prioritisation literature [24–26] and supplemented with input from members of the Assisted Dying Research Network.

#### Assisted dying research network

The survey was developed using a process of collaborative design with members of the Assisted Dying Research Network, [34] an international and independent group established to facilitate research on AD in NZ. The aim of the Assisted Dying Research Network is two-fold: firstly, to establish an independent, multi-disciplinary group of academics, clinicians, hospital, community and hospice senior managers and executive leaders to discuss issues arising from the introduction of AD. Secondly, to facilitate research collaborations that will facilitate the conduct of timely and high-quality AD research in NZ. The Assisted Dying Research Network endeavours to ensure that members hold a variety of views regarding AD.

The purposeful establishment of a methodologically and culturally diverse collaborative network was a way of quality-checking our approach to this study. Members of the Assisted Dying Research Network hold differing views on assisted dying, but seek the same goal: to provide an evidence base to inform law, policy and practice regarding AD and the End of Life Choice Act [34]. The Assisted Dying Research Network comprises researchers and clinicians from palliative care, public health, primary health, psychology, sociology, law, oncology, nursing, psychiatry, and Māori studies, as well as lay people, hospice management, and the Ministry of Health Registrar for AD.^1^

A four-hour online hui was attended by 20 Assisted Dying Research Network members on 8 February 2022. Members were assigned to disciplinary groups (clinical, legal, Māori-centred research, qualitative, quantitative). Each group was asked to discuss their views on the key priorities for AD research in Aotearoa NZ, including how and when such research should be conducted. The exercise was framed as follows:

This research is focused on assisted dying in New Zealand. We want to identify both short- medium- and long-term priorities. The target audience for the research outcomes are policymakers, practitioners, the public, and end-of-life researchers. The beneficiaries of this research are the public at large, practitioners and people eligible for assisted dying.

Relevant criteria for research prioritisation includes:

- Greatest public health benefit
- Relevance for patients and providers
- Likelihood of funding
- Cost of research
- Feasibility
- Relevance for the Act’s 2024 review and timeframe of research
- Te Tiriti o Waitangi principles
- NZ Health Research Prioritisation Framework Attributes: why in NZ, mana tāngata (defined as the importance and value of Māori knowledge and ways of knowing and doing research), excellence, impact and equity [27–31, 40].

#### Research item construction

The small group ideation exercise generated almost 100 research ideas, many of which overlapped. Advice from network members with quantitative expertise and practical perspectives from clinicians suggested that 15 topics was the optimal number of research items for the survey. To reduce the number of research ideas for the purposes of the survey, we identified and removed those topics which could not be answered before the Act’s 2024 review. The remaining research topics were then synthesised and distilled down by a core group of the Assisted Dying Research Network (first three authors). This process was informed by the international and NZ-specific literature on research prioritisation criteria and AD research literature, [27–31, 40] and aimed to ensure the final 15 items were representative of the groups’ suggestions. The survey was piloted with Assisted Dying Research Network members, with feedback resulting in a number of open-ended questions being removed. The amended survey was distributed to health professionals via health professional organisations.

#### Survey format

Using a scale of 0-100, with 100 being the most important, respondents were asked to rate each research item. A rating approach was favoured over a ranking or consensus methodology by the Assisted Dying Research Network who felt that a Delphi analysis similar to Rodgers et al. [25] would be too onerous for HCPs given the impact of COVID-19 on the health care sector at that time. Respondents were asked whether there were any additional areas of importance for research not included in the items provided.

To collect information about current knowledge and experience with AD, respondents were asked to rate their understanding of AD and the new law on a 5-point Likert scale (0 – No understanding, to 5 – Excellent understanding). Respondents were asked how well their organisation is prepared for managing AD, with responses scored on a 10-point (0 – nothing has been done to 10 – my organisation is excellently prepared). Respondents who had first-hand experience of AD were asked to rate their confidence in the procedure on a 10-point scale (0 – I was unconfident in the process to 10 – I was confident in the process). All respondents were invited to provide further comments on their experience with AD.

#### Recruitment

The stakeholder group targeted in this study were HCPs across a range of disciplines. They were chosen because they are in a unique position to provide insights into the most relevant issues related to AD which would contribute to safe, equitable and high-quality care at the end of life. Rodgers et al. observed that “while researchers may be able to identify gaps in the evidence, they may not be best placed to determine which areas are most urgently in need of further research.” [25].

A contact list of individual stakeholders, as well as relevant organisations (e.g., medical colleges, primary healthcare organisations, professional bodies, Māori health organisations, disability and social care organisations, aged care organisations, and other relevant groups such as pro and anti-AD advocacy groups for their health professional members) was compiled by Assisted Dying Research Network members. People / organisations were sent an invitation to participate / circulate the study via email, along with a participant information sheet. Consent was incorporated into the Qualtrics survey. Respondents were given 10 days to complete the survey. To encourage responses, there was a prize draw (four $50 supermarket vouchers) that they could choose to enter.

### Interview study

At the end of the survey, respondents could indicate their willingness to participate in an interview. The email addresses of those willing to be interviewed were collected in a secondary survey to remain separate from their survey responses. Recruitment was supplemented with targeted invites to general practitioners (n = 3) and palliative care physicians (n = 2) to ensure these groups were represented.

The same interviewer (lead author) conducted 16 individual interviews and one focus group with 10 people from one organisation. The purpose of this study was to delve deeper into research priorities and experiences with AD. See interview guide supplementary file 3. Interviews were semi-structured, conducted and recorded via Zoom. The interviews were professionally transcribed, and transcripts were offered to interview participants to review. On average interviews took one hour in duration (range 30–75 min). A $50 supermarket voucher was provided in appreciation for participation.

### Data analysis

For the quantitative data, descriptive statistics were generated using Qualtrics. Qualitative data were analysed using NVivo and a process of thematic analysis as outlined by Braun and Clarke undertaken [35]. Initial codes were generated using an inductive approach and organised into themes and subthemes by the three lead team members (first, second, third authors). For rigour, the final thematic framework was checked with a small group from the Assisted Dying Research Network (fourth, fifth, sixth authors).

## Results

### Survey respondent characteristics

One hundred and forty-three respondents began the survey. Twenty-four respondents (16.8%) were excluded from the data because they did not complete the Research Priority ranking exercise within the survey – from which our results are drawn. In total, 119 respondents completed the survey. The median completion time for the survey was 7.1 min. Demographic information for survey respondents can be found in Table 1.

**Table 1:** Participant Demographics

Category	Sub-Category	Frequency (N)	Percent (%)	Frequency (N)	Percent (%)
		Survey Participants		Interview Participants	
Gender					
Wāhine/Woman		90	76.3	19	73.1
Tāne/Man		26	22	7	26.9
Another gender		1	0.85	-	-
Prefer not to answer		1	0.85	-	-
Ethnicity					
New Zealand European		91	70	16	61.5
Māori		12	9.2	1	3.8
Other European		11	8.5	7	26.9
Other Ethnicity		9	6.9	1	3.8
Indian		2	1.5	-	-
Other Asian		2	1.5	-	-
Chinese		1	0.8	3	11.5
Samoan		1	0.8	1	3.8
Latin American		1	0.8	-	-
*Note: Participants could provide multiple self-identified ethnicities*					
Age					
25–34		6	5.2	2	7.7
35–44		25	21.7	4	15.4
45–54		30	26.1	9	34.6
55–64		42	36.5	7	26.9
65–74		9	7.8	3	11.5
75+		3	2.6	1	3.8
Sector					
Healthcare		102	76.1	26	100
Subsector					
*Secondary Care*		*42*	*41.2*	*4*	*15.4*
*GP/Primary Care*		*21*	*20.6*	*4*	*15.4*
*Community Care*		*18*	*17.6*	*-*	*-*
*Hospice*		*12*	*11.8*	*14*	*53.8*
*Aged Residential Care*		*11*	*10.8*	*3*	*11.5*
Role					
*Medical Doctors*		*37*	*35.2*	*15*	*57.7*
*Nurse Practitioners*		*21*	*20*	*2*	*7.7*
*Registered Nurses*		*18*	*17.1*	*10*	*38.5*
*Management*		*15*	*14.3*	*8*	*30.8*
*Other*		*8*	*7.6*	*3*	*11.5*
*Mental Health Practitioners (Psychiatry/Counselling)*		*4*	*3.8*	*-*	*-*
*Māori Health*		*2*	*1.9*	*-*	*-*
Academia		12	9	-	-
Professional		9	6.7	-	-
Other		8	6	-	-
Role					
*Retiree*		*2*	*25*	*1*	*3.8*
*Governance*		*2*	*25*	*-*	*-*
*Health Navigator*		*1*	*12.5*	*-*	*-*
*Educator*		*1*	*12.5*	*-*	*-*
Government		2	1.5	-	-
Prefer not to answer		1	0.8	-	-
*Note: Participants could provide specific information about their subsector and role & could work across multiple sectors*					

Note: All % rounded to 1dp.

Most respondents rated themselves as having a ‘moderate − 3’ to ‘good − 4’ understanding of AD and the End of Life Choice Act, where 5 is excellent, *M =* 3.88, *SD* = 0.80. Of those eligible to complete Ministry of Health AD training, 49% had completed at least one module. Respondents gave a wide range of ratings on their organisations’ level of preparedness, *M* = 5.79, *SD* = 2.76. Scores trended towards ‘moderately well-prepared’, however, the scores ranged from 0 to 10 (reflected in the high standard deviation. Almost a third (35 respondents) had first-hand experience with AD at the time of the survey. Confidence in the AD process trended towards the higher end of the scale, *M* = 6.76, *SD* = 2.43. However, the confidence scores ranged across the scale (0–10).

### Interview and focus group participant characteristics

Demographic information for interview participants can be found in Table 1. For the one-on-one participants, the average health-related work experience was 19 years, ranging from 4.5 years to 38 years. For focus group participants, their experience ranged from 4 months to 18 years. The survey respondents represented almost all districts of NZ whereas the interviewees were mainly from the North Island where more people reside.

### Stakeholders’ priorities for assisted dying research

Survey data for the research priority rating indicated that respondents thought all 15 items were important, some moderately more so than others. However, there were high levels of variation within each item, indicating a lack of consensus on importance. Data is presented in Table 2. Qualtrics was used to compute the average importance score out of 100 for each area. The highest rated area of importance for research was ‘*Effectiveness of the safeguards in the Act to protect people’* (*M* = 84.5, *SD* = 23.7) and the lowest rated item was ‘*Experiences of non-provider (e.g., admin, cleaning) staff where assisted dying is being provided’* (*M =* 62.4, *SD* = 26.8).

**Table 2:** Average importance score for each research item

Research Priority Area	Average Importance Score
1. Effectiveness of the safeguards in the Act to protect people	*M* = 84.5, *SD* = 23.7
2. The relation with and impact of assisted dying on palliative care	*M* = 83.4, *SD* = 21.8
3. Experiences of people and whānau/family choosing and practitioners providing assisted dying	*M* = 82.8, *SD* = 20.7
4. Views of people from disability communities towards assisted dying	*M* = 80.7, *SD* = 23.1
5. Barriers to individuals exercising their legal right to request assisted dying	*M* = 80.1, *SD* = 21.3
6. Tikanga Māori (custom) and kawa (protocols) and assisted dying	*M* = 79.7, *SD* = 24.2
7. Māori engagement with assisted dying	*M* = 79.6, *SD* = 23.5
8. Experiences of people and providers when assisted dying applications are declined	*M* = 79.2, *SD* = 22.4
9. Evaluation of health practitioner and assisted dying provider training	*M* = 78.8, *SD* = 21.4
10. Impact on structurally disadvantaged groups	*M* = 78.3, *SD* = 24.2
11. Health practitioners’ (including assisted dying providers) interpretation of the eligibility criteria outlined in the End of Life Choice Act	*M* = 77.6, *SD* = 25.1
12. Analysing Ministry of Health data on assisted dying engagement and characteristics of people using assisted dying services	*M* = 73.1, *SD* = 25.3
13. Timeliness of service provision	*M* = 72.9, *SD* = 27.2
14. Stigmatisation of those involved in the provision of and use of assisted dying services	*M* = 70.1, *SD* = 28.1
15. Experiences of non-provider (e.g., admin, cleaning) staff where assisted dying is being provided	*M* = 62.4, *SD* = 26.8

Note: all numbers have been rounded to 1 dp.

Interestingly, all items had a high standard deviation (SD) (ranging from 20.7 to 28.1). Larger standard deviations indicate that data is spread across the ratings scale. This suggests that for each item there was a sizable range of ratings, indicating a lack of consensus within ratings. Taken together the moderate variation between mean ratings of each item and the high variation within each item implies that all of the items were important to respondents, and while some averaged higher ratings, there were no items that were considered unimportant. This was confirmed by respondents in the free text comments where some stated that all were important areas for future research.

Of note, the experiences of non-providers caring for AD patients was a prominent topic in the interviews, yet it had the lowest mean in the survey. The item in the survey used non-clinical staff as examples of non-providers i.e. staff who are proxy to AD but not directly involved in the provision of AD. HCP stakeholders may not have considered the experiences of administrative or cleaning staff as highly important and these groups did not participate in the research themselves. The specificity of the survey item as compared to the broad possible interpretations of the term in the interviews is a likely explanation of the difference in prominence. When synthesising the original research priorities with the qualitative data from survey comments and interviews this issue was considered and the theme ‘experiences at the bedside’ includes a broad definition of ‘non-provider’. As well as including non-clinical staff in this definition, we also categorised stakeholders’ comments about those who are not permitted by law to provide AD services (e.g. nurses and healthcare assistants) as well as healthcare practitioners who chose not to provide AD on the basis of conscience to this item. Also included under this umbrella term are those who are non-providers because their employer restricts participation. We note that a limitation of this broad definition is that it does not permit discerning the different bases reasons for being a ‘non-provider’, hence our call below for future research to examine this.

### Research priorities from the field

The question ‘what other aspects of assisted dying, if any, do you think are important to research?’ produced 49 suggestions from the 119 survey respondents and many more from interview participants. The concluding question (‘do you have any further comments, concerns, or information about assisted dying that is important for researchers to take into account?’) produced many other comments that have also informed the research priorities.

Some existential/spiritual research topics were suggested, while others were experiential, pragmatic and clinical in nature. Research suggestions by stakeholders were grouped into 16 areas, including: whānau/family experiences; capacity; AD providers; people seeking AD; conscientious objection; EoLC Act; palliative care; socio-cultural; death event; AD service provision; uptake; aged care; decision-making; non-providers; health workforce. To further refine these areas, and supplemented by the research suggested in the interviews, we categorised the research topics within the following themes: (1) overarching context of the health system; (2) experiences at the bedside; (3) medico-legal issues; (4) impact of AD which is ‘a different kind of dying’; (5) on the day of dying; and (6) the overall effectiveness of the AD system. See Table 3 for definitions and examples. Interestingly, the *ab initio* research priorities that we asked participants to rate the importance of, also largely fell within these broad themes (see Table 3). As well, some research priorities could be assigned to multiple themes e.g. experiences of people and whānau/family choosing and practitioners providing assisted dying pertains to three themes: experiences at the bedside, ‘a different kind of dying’, and on the day of dying. Table 3 displays the synthesis of the research priority rankings from the survey and the thematic analysis of survey comments and interview data. This presentation of data provides an overview of six key thematic areas for research, with specific thematically linked research questions (15 with importance rankings), and many further research questions directly from HCPs (with exemplar quotes for additional context). This table is a map for future stakeholder-directed AD study within New Zealand which we encourage other researchers to use.

**Table 3:** Research Priorities – Survey Rating Scores & Additional Qualitative Input from participants

Theme	Description of theme	Corresponding Research Priority Items from Survey, Ranking & Importance Scores	Research questions identified by survey respondents and interview participants	Exemplar quotes about research priorities from survey respondents
Overarching context of the health system	Research was necessary in relation to the contextual factors, namely the stretched health system, the inequities that already exist, and the relationship between hospice/palliative care and AD.	2. The relation with and impact of assisted dying on palliative care (*M =* 83.4, *SD* = 21.8) 4. Views of people from disability communities towards assisted dying (*M* = 80.7, *SD* = 23.1) 7. Māori engagement with assisted dying (*M* = 79.6, *SD* = 23.5) 10. Impact on structurally disadvantaged groups (*M* = 78.3, *SD* = 24.2)	• What effect has AD had on attitudes towards death and dying and openness towards discussing dying over time for both health professionals and the public? • Is bias affecting equitable access to AD for various groups? • Whether people requesting AD had access to high-quality palliative care, was it routinely being offered to those not accessing it, the effectiveness of palliative care to relieve their suffering and how often accessing palliative care relieved the desire for AD?	“Understanding why people choose the path or [sic] assisted dying is imperative. Separating End of Life Care services from Assisted Dying and understanding what both have to offer is imperative. An example of this, is that some people who choose assisted dying are afraid of suffering but have no true understanding of how the palliative care process reduces suffering at the end of life.”
Experiences at the bedside	Understanding the experiences of patients and families receiving AD services and HCPs, either providing AD or caring for people receiving AD (i.e. non-providers)	3. Experiences of people and whānau/family choosing and practitioners providing assisted dying (*M =* 82.8, *SD* = 20.7) 8. Experiences of people and providers when assisted dying applications are declined (*M* = 79.2, *SD* = 22.4) 15. Experiences of non-provider (e.g., admin, cleaning) staff where assisted dying is being provided (*M =* 62.4, *SD* = 26.8)	• What communication skills are required to respond to AD requests for all HCPs? • How to manage the AD process sensitively, especially when a person was found ineligible and how best to support patients and to hand back over care to their usual clinicians. • What the legal prohibition on raising AD means for clinical practice as well as patients being informed about their end-of-life options? • What effect is confidentiality (and ‘secrecy’) having on staff across all sectors? • What are the views of doctors’ post-legalisation, in particular oncologists who have not been surveyed (since most AD patients have cancer)? • What is the role of doctors’ religion in their responses to AD? • Is AD having any impact on staff burnout? • How to provide culturally competent care?	“Investigating the beliefs, attitudes, perspectives and behaviours of health professionals within health services (primary, tertiary health care and specialist health care) towards treating patients/whānau who has an AD in place (poor responses by health professionals can minimise and disrespect the decision making and treatment of the AD person/whānau). Do strong negative attitudes affect the perceptions of health professionals and compromise the treatment the AD person and whānau receive?”
Medico-legal issues	Stakeholders discussed the importance of researching how the legal criteria for eligibility are/should be applied.	1. Effectiveness of the safeguards in the Act to protect people (*M* = 84.5, *SD* = 23.7 5. Barriers to individuals exercising their legal right to request assisted dying (*M* = 80.1, *SD* = 21.3) 11. Health practitioners’ (including assisted dying providers) interpretation of the eligibility criteria outlined in the End of Life Choice Act (*M* = 77.6, *SD* = 25.1	• How are AD providers interpreting the End of Life Choice Act eligibility criteria? • Who is qualified and what training is necessary/sufficient/adequate for assessing capacity? • What clinical advice and clinical tools do clinicians use to assess eligibility/prognosis? • What processes are/should be in place where a patient’s primary team disagree with the AMP/IMP assessment? • How is/should “unbearable suffering” be determined/defined? • Tension between respect for the confidentiality and privacy of the patient with the desirability of ensuring the extended/primary care team are aware of the patients end of life plans (this may be less of an issue if/when AD becomes more normalised). • What processes should be in place for disagreeing with eligibility assessments e.g. raising concerns, making complaints. • To what extent should prognostication be ‘narrow’ or ‘holistic’ – what approaches to “likely to die” should be adopted? • What should the law be and how do we decide?	“Capacity assessment is a difficult area requiring expert assessment, and I don’t think we know what the capabilities of those providing assisted dying are in this area, so research into how and when practitioners are assessing capacity would be important” “Aspects where law is deficient: a) inability to use Act due to lack of process for advance directives e.g. dementia, and patients who lose their ability to think at the last minute due to their advancing disease b) the place of nurse practitioners in the process - nurses are integral to the process and more power and representation should be given to the nurse practitioners who are involved”
‘A different kind of dying’	This theme yielded an array of research questions focused on the impact of AD being a different type of death.	3. Experiences of people and whānau/family choosing and practitioners providing assisted dying (*M =* 82.8, *SD* = 20.7) 6. Tikanga Māori (custom) and kawa (protocols) and assisted dying (*M* = 79.7, *SD* = 24.2) 14. Stigmatisation of those involved in the provision of and use of assisted dying services (*M =* 70.1, *SD* = 28.1) 15. Experiences of non-provider (e.g., admin, cleaning) staff where assisted dying is being provided (*M =* 62.4, *SD* = 26.8)	• How does an AD differ from an “ordinary” or non-assisted death? How is it similar? • What is the impact of secrecy on staff/carers? • What role does stigma play in ‘othering’ AD? • What counts as a good death? • What impact does AD have on the bereavement experience? • How do providers rationalise and navigate being an AD provider? How do providers manage their role i.e. navigate personal and family dynamics? • What are the experiences of providers of this different kind of dying, how are they perceived by others and is there any impact on service provision? • How have perspectives about AD shifted now that it is legal? • How does grief differ and how is it similar for AD? • How to provide bereavement support for a patient’s family?	"The effect of assisted dying on those left behind, who had no prior knowledge that assisted dying was being sought or had been actioned. How does assisted dying affect the bereavement process, with/without prior knowledge that assisted dying had been activated.”
On the day	Research under this theme pertained to expectations of providers, how patients were curating their deaths and the administration of drugs.	3. Experiences of people and whānau/family choosing and practitioners providing assisted dying (*M =* 82.8, *SD* = 20.7) 6. Tikanga Māori (custom) and kawa (protocols) and assisted dying (*M* = 79.7, *SD* = 24.2)	• Does the patient and family expect the AMP to act as a “celebrant” or as a “technician” or is it not important? • Is it important that the original AMP returns for the assisted death itself, having established a ‘relationship’, or is it more important that the event occurs when and where requested by the patient and family irrespective of the operator? • Should the AMP feel able to negotiate with the patient and family as to the timing etc or should that be considered sacrosanct? • What new death rituals are emerging? e.g. death plans • What was the quality of death? • Were there any administration issues? • Etiquette – how to speak to patient/family?	“The experiences of the family are obviously very important and perhaps research directed at giving AMPs specific guidance on how to carry out their duties on the day of death: eg drawing up the medication remote from the patient, whether a long extension line on the IV should be used so that the AMP can be away from the patient allowing family to be able to be the focus of attention, whether family want to be alone with the patient immediately after administration or whether they would prefer the “security” of the AMP’s presence for those few minutes until the patient is declared deceased, how to "do the paperwork" in a small crowded apartment surrounded by strong emotional family where one no longer really belongs. Practical suggestions that could improve the consistency of service”
Overall effectiveness of the AD system	Topics about how the AD was being implemented across the health sector and the variety of implementation models in particular in Aged Residential Care, the role of conscientious and institutional objection, workforce and training issues, and the Ministry of Health reporting data.	1. Effectiveness of the safeguards in the Act to protect people (*M* = 84.5, *SD* = 23.7) 5. Barriers to individuals exercising their legal right to request assisted dying (*M* = 80.1, *SD* = 21.3) 9. Evaluation of health practitioner and assisted dying provider training (*M* = 78.8, *SD* = 21.4) 12. Analysing Ministry of Health data on assisted dying engagement and characteristics of people using assisted dying services (*M* = 73.1, *SD* = 25.3) 13. Timeliness of service provision (*M* = 72.9, *SD* = 27.2)	• What are the access issues for Aged Residential Care (ARC) residents? Are facilities allowing deaths to occur on site, does this breach people’s rights, do living arrangements (independent villa, hospital level etc) affect this or not? How many people are having to transfer elsewhere to die and what is the effect on people and families? How to keep residents and staff safe when they don’t want to care for an AD patient? What are experiences of providers going on objecting facility? Should institutions be able to object, particularly if they receive government funding? • What role do personal beliefs about AD play a role in how an organisation implements AD? • What would help practitioners who are uncertain about participating decide to become a provider? What is the minimum number of providers/patients – i.e. a reasonable caseload, especially as demand increases? • Whether access is equitable across groups, the timeliness of access, what type of palliative care AD patients were accessing, whether it was meeting their needs, • Why did people seek AD? • How does NZ data compare to overseas jurisdictions?	“How staff who work in organisations with a conscientious objection reconcile if they have a personal opinion that supports assisted dying?”

Note: all numbers have been rounded to 1 dp.

## Discussion

This study identifies a comprehensive map of research priorities produced by stakeholders in a healthcare system where AD has recently become legal. This context means that our study differed in terms of stakeholder groups, and methodology from the Delphi study conducted by Rodgers et al. [25] Rodgers et al. included AD campaigners, patients and carers, in the context of a country still debating whether AD should be legalised. In contrast, our study had a significant focus on the delivery of AD and on health care providers. Furthermore, our items were generated by the Assisted Dying Research Network (including a lay person), whereas Rodgers et al.’s stakeholders produced the material for the Delphi study. Despite these differences, similar themes were apparent in our findings. One exception was Rodgers et al.’s findings about arguments for and against AD. Arguments for or against AD did not feature in our stakeholders’ comments, though their views on AD likely informed what they ranked as important areas of research. Even when disagreeing with the legislation, the comments were focussed on needing to work with the new law to ensure it was operating safely. We posit that the debate regarding legalising AD has largely concluded in NZ because of the national binding referendum which took place prior to the legalisation.

Early exposure to AD, whether directly providing AD or not, enabled participants to reflect on what research is necessary to monitor the provision of AD and inform the development of law, policy and practice. The high variation in how prepared and confident respondents rated themselves indicates inconsistencies across the health system; research into these inconsistencies and their source is worthwhile. Using free text survey questions in addition to the ranking exercise produced rich insights into clinical practice promoting the safety and well-being of all people involved or affected by AD. While the two techniques produced differing results, they complement and expand upon each other.

Another way of conceptualising the study findings is to consider how respondents weigh up the various items they rated. By this, we mean exploring what values underpin their views. A systematic review of health professionals’ perspectives on AD implementation barriers and facilitators identified that personal and professional values act as both barriers and facilitators and influence how HCPs prioritise clinical issues [36]. In Australia, HCPs’ attitudes towards AD are informed by their beliefs, emotions, education, and strength of religious beliefs, but not knowledge of AD [37]. The authors of these two studies [36, 37] advocate for more research with HCPs and stakeholders regarding ‘safeguarding’ and assessing ‘capacity’ and for HCPs’ reflexive practice about the influence of values and feelings on clinical practice [36, 37]. Safety is a priority for respondents as referenced by items 1, 4 and 10 (see Table 2). Access is referenced by items 5, 9, 11, 13, 14. Balancing safety and access is an important policy goal of any AD system [1, 2, 38, 39]. Access must be facilitated for eligible people, while restricting access to those who are not. That the highest rated item is safety vis-a-vis protecting people and not ‘does the Act support patient need and choice as it is intended?’ points to the uncertainty that all HCPs may be experiencing as the people appointed as the gatekeepers of AD, and thus a new form of responsibility for patients, over and above their beliefs around access. Or it may reflect the personal views of stakeholders regarding whether AD should be available or not. Equity underpins items 2, 7, 12. Equity is an important consideration for the NZ health and research systems to meet Te Tiriti obligations [27, 40].

In the AD context, equity refers to access to, and utilisation of, AD services for all communities. There is growing evidence in the palliative care literature of the inequities in access to end of life care for some groups [41, 42]. Understanding whether unmet end-of-life care needs are driving requests for AD in some groups is needed [43]. The well-being of patients, families, whānau, providers and non-providers underpins items 3, 6, 8, 15. These values are important given that the Ministry of Health states that the service “aims to be equitable and ensure people and their family/whānau are at the centre, that there are effective safeguards as set out under the law, and that the service is accessible to those who meet the eligibility criteria” and also aims to be culturally safe for Māori [27, 44, 45].

### Research road map

The findings from this study are now being used to inform a research programme being developed by the authors. The results of the survey and interviews point to both large-scale overarching research that aims to map existing practice and identify wider issues, as well as the importance of smaller, targeted research projects that address key areas of concern for NZ.

The research prioritisation exercise directs us to focus on safety and access research. Safety means the AD system operates effectively to discern eligibility and exclude those not eligible in a timely manner. It also means AD is culturally, psychologically, and ethically safe for all involved. The Act contains safeguards to ensure the person requesting AD is acting of their own volition and is competent to make such a decision. Theoretical critiques of the legislation are emerging, highlighting the tensions in interpreting the Act, scrutinising the scope of eligibility, safeguards, and people who are excluded [1, 46]. Empirical research is needed to understand if the safeguards, while having individual merit, act as barriers to legally available AD when applied together [47–49]. Whether access to AD and quality information and services is equitable across social groups is currently unknown.

Providing an evidence base to inform the 2024 review of the operation of the Act requires extensive data collection and analysis to identify safety and access issues and areas for improvement in the provision and oversight of AD services [1, 2]. Given our focus on what research is most urgent, impactful and feasible (see criteria above), long-term research priorities were not included in the 15 items selected for the survey. However, stakeholders commented on longer-term research involving cultural, existential, and societal issues (see Table 3). These will form an important aspect of future research.

A particular research priority is the impact on, and experiences of, Māori regarding AD, given the known health inequities for Māori that is rooted in colonisation, institutional bias, and inequities in access to, and quality of, healthcare [50]. Exploring what the safety of an AD service looks like for Māori (and other countries with indigenous or ethnic minority groups) is an important area of research. While studies have contributed to the understanding of Māori perspectives towards AD before it became legal, [17, 18] further research is needed to appreciate how Māori are responding to AD post-legalisation. A further project that may not seem important according to the research prioritisation results is the experiences of non-clinical staff. These staff can receive initial enquiries about AD so understanding their views, experiences and what training they require is warranted. We are not aware of any studies that examine this cohort.

### Limitations and strengths

A study limitation is the adapted research prioritisation method used which meant the items for ranking were produced by researchers and clinicians, rather than stakeholder groups. However, to compensate for this, the free text survey questions asked respondents to suggest additional research areas. This was an important addition to the survey as it gave respondents and opportunity to elaborate on the items for ranking. A benefit of having the research items for ranking determined by subject experts meant that there was no duplication of topics with existing research and major research gaps able to be incorporated. A further limitation is that the methodology used did not force participants to prioritise research items relative to each other, permitting them to respond that all or most of the items were important. While a ranked list emerges from this method, future AD research priority research could consider a forced ranking exercise which would demonstrate the items’ relative importance.

In terms of the sample, almost a third of respondents had first-hand experience with AD, which is significant given the small size of the AD provider population in NZ. The overrepresentation is both a strength, in that we captured people with some experience of AD, and a limitation in that we used a broad description and thus cannot differentiate how directly involved they were in AD. As noted above, similar issues also arise in relation to the category of ‘non-provider’ as our approach does not report on the different cohorts that are captured in this category and their potentially different views on AD. Further, the ethnicity of the stakeholders do not reflect the NZ population. However, the high proportion of women and people aged over 40, as well as the low proportion of Māori and Pacific people, reflects ongoing trends within the health workforce [51].

The timing of this research is both a limitation and a strength. AD had been legal for only five months at the time of the study, meaning that we captured issues stakeholders were experiencing in the early stages of implementation. These early experiences informed their views on what research is necessary. However, the timing of the survey also meant its distribution and capacity to respond was impacted by COVID. This may have limited the number of respondents and meant only those with an interest responded.

Although the authors agree public involvement in priority setting is important, we focused on health professional stakeholders and did not include patients, caregivers or the public. This resulted in omitting crucial stakeholder groups’ perspectives that is likely to have produced a differently ranked list and other suggestions for areas of research. Other studies are underway with these groups and we have plans to involve them in future research, borne out of this survey.

## Conclusion

As identified by stakeholders and informed by researchers, this article outlines many areas for future research and the specific questions that comprise each area. AD research was described by stakeholders as vital because it would inform best practice for supporting patients, families, providers and non-providers in what is a new and evolving clinical service. The findings and research agenda may be useful for other jurisdictions that have recently legalised AD, or are planning to implement an AD service.

The HCPs participating in this research agenda setting exercise self-reported they were an informed cohort. They provided rich insights, identifying numerous research questions and issues. Given the expressed emphasis on safe, equitable, and effective provision of AD services for NZ patients, families and whānau, understanding how this new health service is functioning during the early stages of its implementation is crucial. Conducting the research outlined in this paper is warranted to address one of the most significant services to be introduced into NZ’s health care system.

## Electronic supplementary material

Below is the link to the electronic supplementary material.

Supplementary Material 1

Supplementary Material 2

Supplementary Material 3

## Data Availability

The datasets generated during and/or analysed during the current study are not publicly available due to participant confidentiality but may be available from the corresponding author on reasonable request.

## Footnotes

1 The functions of the Registrar AD include reviewing the relevant forms that must be lodged at each step in the process with each AD case to check the processes followed comply with the Act before any medication is dispensed.

## Abbreviations

AD: assisted dying
AMP: attending medical practitioner
ARC: aged residential care
EoLC Act/the Act: End of Life Choice Act 2019
GP: general practice/general practitioner
HCP: healthcare professional
IMP: independent medical practitioner
NZ: Aotearoa New Zealand
SD: standard deviation
UK: United Kingdom
