# Supplementary material for: What do health care professionals want to know about assisted dying? Setting the research agenda in New Zealand
Source: BMC Palliat Care. 2023 Apr 10;22:40. doi: 10.1186/s12904-023-01159-8 (PMC10084592; doi:10.1186/s12904-023-01159-8)
Supplement: Supplementary file 1 — Supplementary Material 1 [file 12904_2023_1159_MOESM1_ESM.docx]

Supplementary file

Figure 1: Simplified assisted dying application process and safeguards

| Person requesting Assisted Dying | Attending Medical Practitioner (AMP) | Independent Medical Practitioner (IMP) | Psychiatrist | Registrar (Assisted Dying) |
| --- | --- | --- | --- | --- |
| Preliminary request  (s 11) ***** |  |  |  |  |
|  | Completes safeguard checks (s 11) ** |  |  |  |
| Request confirmed  (s 12) |  |  |  |  |
|  | First opinion on eligibility  (s 13) *** |  |  |  |
|  |  | Second opinion  (s 14) |  |  |
|  |  |  | Third opinion if AMP/IMP not satisfied of person’s competence  (s 15) |  |
|  | Informs person if not eligible (s 16) or eligible (s 17)  **** |  |  |  |
| Choose date & time for administration of medications (s 18) and method of administration (s 19) |  |  |  |  |
|  |  |  |  | Checks s 11-18 complied with  ***** |
| Self-administer medication  ****** | Administration of medication (s 20) #  ****** |  |  |  |
|  | Report death to Registrar (Assisted Dying) (s 21) # |  |  |  |
| Legend:  Form sent to Registrar/a copy of the form is sent to AMP and IMP  Patient can change mind at any time  Any pressure suspected by AMP or IMP, the process must stop and be reported to the  Registrar  # An attending nurse practitioner can also administer the medication and complete the death report.  Safeguards  * A HCP must not, in the course of providing any health service, initiate any discussion that is in substance about AD, or make any suggestion that is, in substance, a suggestion to undergo AD (s10).  ** After a person raises the issue of AD, the AMP must give specified information to the person before an application for a formal assessment may be made (s6). This includes: giving the prognosis of the terminal illness and explaining the irreversible nature of AD. The AMP must also ensure the individual knows about other options for end of life care. Additional obligations include encouraging the person to discuss with family (but ensuring they know they are not obliged to do so) and ensuring they know they can change their mind at any stage. The AMP must do their best to ensure the patient’s wish is expressed free from pressure (e.g. by conferring with other HCPs involved, and members of their family).  *** If at any time the AMP suspects (on reasonable grounds) that the person is not expressing their wish free from pressure, they must take no further action and notify the Registrar (s24).  **** Specific eligibility criteria must be met, including mental capacity which cannot be presumed (s5(1)). Further, a person cannot be an eligible person by reason *only* that they are suffering from: any form of mental disorder or illness; has a disability of any kind; or is of advanced age (s5(2)).  ***** AD must not be performed *unless* the Registrar AD gives notice that they are satisfied that all the requirements of the Act have been complied with (s20(1)).  ******AD requires a competent, contemporaneous request by the eligible person. Advance directives for AD are not permitted (s33). Welfare Guardians/Enduring Power of Attorney’s have no power to make decisions if a person is not competent (s34, schedule). | | | | |
