# Supplementary material for: What do health care professionals want to know about assisted dying? Setting the research agenda in New Zealand
Source: BMC Palliat Care. 2023 Apr 10;22:40. doi: 10.1186/s12904-023-01159-8 (PMC10084592; doi:10.1186/s12904-023-01159-8)
Supplement: Supplementary file 2 — Supplementary Material 2 [file 12904_2023_1159_MOESM2_ESM.pdf]

Intro and Consent

Thank you for your interest in this research. This survey is being disseminated by a new, independent, and multidisciplinary assisted dying research network, comprised of members with a variety of views.

The End of Life Choice Act (2019) legalises assisted dying from 7 November 2021. This means a range of new decisions for some people and their whānau to make and new roles for health practitioners.

The purpose of this survey is to capture the views of assisted dying stakeholders to help us determine the direction of future research. We are also asking demographic questions and closed ended questions about what is happening in practice. You don’t have to answer any questions you don’t feel comfortable answering.

Your participation is voluntary and will take 5-10 minutes. We greatly appreciate you taking the time to respond as we know health and social care sector workers are incredibly busy with the Omicron outbreak currently.

The data collected will be securely stored (password access only) on the principal investigator Dr Jessica Young's secure server provided by Victoria University of Wellington. By participating in this survey, you are providing us with consent to use the information you have shared with us. All responses will be confidential. We will be working with aggregated data to ensure responses are not able to be identified. Once you submit the survey, it will be impossible to retract your answer.

The results of the project may be published in research articles, media articles, and conference presentations. You can opt in to receive a copy of the research article and enter the prize draw (4 x $50 supermarket vouchers) at the end of the survey – your email address will be collected and kept separately from your response.

Continuing with this survey implies consent. I understand and consent to participate:

- Yes
- No
- This survey is not relevant to me (if so, please explain why in the text entry field below)

Demographics

Thank you for agreeing to take part in the survey.

This section asks you about your age, gender identity, ethnicity, the region you work from, and your work role.

What is your gender(s)?

- Man/Tāne
- Woman/Wāhine
- Another gender (please specify)
- Prefer not to answer

Which ethnic group(s) do you belong to?
Please select all that apply

- New Zealand European
- Māori
- Other European
- Samoan
- Cook Islands Māori
- Tongan
- Niuean

- ☐ Tokelauan
- ☐ Fijian
- ☐ Other Pacific Peoples
- ☐ Southeast Asian
- ☐ Chinese
- ☐ Indian
- ☐ Other Asian
- ☐ Middle Eastern
- ☐ Latin American
- ☐ African
- ☐ Other ethnicity (please specify) __________
- ☐ Don't know
- ☐ Prefer not to answer

How old are you?

- ○ Under 25 years
- ○ 25 to 34 years
- ○ 35 to 44 years
- ○ 45 to 54 years
- ○ 55 to 64 years
- ○ 65 to 74 years
- ○ 75 years or over
- ○ Prefer not to answer

Which region(s) do you work from?
Please select all that apply

- ☐ National organisation
- ☐ Te Tai Tokerau / Northland
- ☐ Tāmaki Makaurau / Auckland
- ☐ Waikato
- ☐ Te Moana-a-Toi / Bay of Plenty

- ☐ Tūranganui-a-Kiwa / Gisborne
- ☐ Te Matau-a-Māui / Hawke's Bay
- ☐ Taranaki
- ☐ Manawatū-Wanganui
- ☐ Te Whanganui-a-Tara / Wellington
- ☐ Te Tai-o-Aorere / Tasman
- ☐ Whakatū / Nelson
- ☐ Wairau / Marlborough
- ☐ Te Tai Poutini / West Coast
- ☐ Waitaha / Canterbury
- ☐ Ōtākou / Otago
- ☐ Murihiku / Southland
- ☐ Prefer not to answer

Which sector(s) do you work in?
Please select all that apply

- ☐ Academia
- ☐ Health care (please name your place of work e.g. DHB, community) __________
- ☐ Professional
- ☐ Government
- ☐ Other (please specify) __________
- ☐ Prefer not to answer

What is your current role:

____

How long have you been working in that role?

- ○ Less than 2 years
- ○ 2-5 years
- ○ 6-10 years

- 11-15 years
- 16-20 years
- 21-30 years
- 31-40 years
- 41 years or more
- Prefer not to answer

How would you rate your understanding of assisted dying and the new law, the End of Life Choice Act?

- No understanding
- A little understanding
- Average understanding
- Good understanding
- Excellent understanding

Have you completed any of the Ministry of Health assisted dying training modules? (https://www.health.govt.nz/our-work/life-stages/assisted-dying-service/information-health-professionals/training-resources-health-professionals)

- Yes. Please specify which modules below.
- No
- Not sure
- Not eligible to complete training

Research Priorities

Thank you.

Now we'd like to ask you what you think are the most important areas for us as researchers to investigate. We will use this information to help us set research

directions in the short to medium-terms.

Please consider the following 15 assisted dying research areas that our independent network identified. Use the sliding scale to how rate how important you think each research area is.

0= Not important at all, 100= Extremely important

	Not at all important		Slightly		Moderately		Extremely important				
	0	10	20	30	40	50	60	70	80	90	100
Barriers to individuals exercising their legal right to request assisted dying											
Health practitioners' (including assisted dying providers) interpretation of the eligibility criteria outlined in the End of Life Choice Act											
Experiences of non-provider (e.g. admin, cleaning) staff where assisted dying is being provided											
Stigmatisation of those involved in the provision of and use of assisted dying services											
Māori engagement with assisted dying											
Analysing Ministry of Health data on assisted dying engagement and characteristics of people using assisted dying services											

	Not at all important		Slightly			Moderately			Extremely important		
	0	10	20	30	40	50	60	70	80	90	100
The relation with and impact of assisted dying on palliative care											
Tikanga Māori (custom) and kawa (protocols) and assisted dying											
Effectiveness of the safeguards in the Act to protect people											
Impact on structurally disadvantaged groups											
Timeliness of service provision											
Evaluation of health practitioner and assisted dying provider training											
Views of people from disability communities towards assisted dying											
Experiences of people and whānau choosing and practitioners providing assisted dying											
Experiences of people and providers when assisted dying applications are declined											

What other aspects of assisted dying, if any, do you think are important to research?

Current Work

Thank you. The next section explores what is currently happening in practice with assisted dying.

How well, to your knowledge, is your organisation prepared for managing assisted dying requests?

Nothing has been done				Moderately prepared			My organisation is excellently prepared				Not Applicable
0	1	2	3	4	5	6	7	8	9	10	☐

Have you had any experiences with assisted dying since it became legally available?

- ○ Yes
- ○ No
- ○ Not applicable

If yes, how would you rate that experiences from a procedural point of view?

I was unconfident in the process										I was confident in the proces
0	1	2	3	4	5	6	7	8	9	10

Do you have any further comments, concerns, or information about assisted dying that its important for researchers to take into account?

Contact Info & Prize Draw

Thank you for taking the time to respond to this survey. Please click 'yes' below to enter your email address for the prize draw. On the next page there is also an option to indicate you would like to receive a copy of the publication and/or you would be willing to be contacted about further research. If you choose to enter your email address it will be kept separate from your response and you will only be contacted for the option(s) you select. Only the immediate research team will have access to your email address and these will be deleted at the end of the project.

If you do not want to provide your email address, please click 'no' below and your response will be recorded.

- Yes
- No

Powered by Qualtrics
