# Supplementary material for: What do health care professionals want to know about assisted dying? Setting the research agenda in New Zealand
Source: BMC Palliat Care. 2023 Apr 10;22:40. doi: 10.1186/s12904-023-01159-8 (PMC10084592; doi:10.1186/s12904-023-01159-8)
Supplement: Supplementary file 3 — Supplementary Material 3 [file 12904_2023_1159_MOESM3_ESM.docx]

**Assisted Dying Stakeholder Interview Guide**

The questions are to be used responsively and flexibly. Questions may need to be tailored for the different groups of stakeholders once we confirm who our interviewees are. The interviewer will monitor the participant throughout the interview to ensure they’re feeling ok and want to continue.

**Opening the Interview**

Whakawhanaungatanga – general conversation, getting to know the person a bit, connecting

If Māori stakeholders are being interviewed, follow the tikanga of the stakeholder

Establish whanaungatanga; introducing self, whakapapa, whānau connections, work etc [can be brief or longer if needed]

If they don’t lead with karakia, mihi etc I would initiate this with them “would you like us to start with karakia?” [karakia to be nominated by the Network’s kaumatua and Māori members]

**Whakataka te hau ki te uru
Whakataka te hau ki te tonga
Kia mākinakina ki uta
Kia mātaratara ki tai
E hī ake ana te atakura
He tio, he huka, he hau hū
Tīhei mauri ora!**

Then follow as per this section…

Is it ok to turn on the recorder now?

Thank you for agreeing to talk with me and signing the consent form.

On the information sheet and consent form there were two options for confidentiality. You can choose to be identified by your actual name and job title or we can keep your identity confidential by using a pseudonym and/or referring to you by your role.

**If participant wants to remain confidential:**

Do you have a pseudonym you’d like to use – or I can assign you one. Are you comfortable being referred to by your role? What title would you like me to use?

As you know from the survey and information sheet, we’d really like to get a sense of what AD stakeholders think are key research areas that researchers should be focussing on to research if the process of AD is safe, accessible, and equitable. We will also ask about how has the legalisation of assisted dying changed the work that you and your organisation does. You don’t have to answer any questions you don’t feel comfortable answering, just say so. Please ask if question is unclear, there’s no right answers, this is about your perspective. We can take a break at any time, just let me know. I’ll take some notes too so I can remember things I would like to ask you more about.

And a final reminder that everything you say is confidential. We won’t use the name of your workplace or any people or services you mention. Do you have any questions before we get started?

**Background information**

To help us describe our sample, can we please begin with your age, gender, ethnicity, role years of experience, sector, region.

**Research questions**

1. Are you aware of any research that is currently being undertaken in the field of AD?
2. What areas of research are important to you when it comes to assisted dying?

2a. If participant needs prompting, in the survey you completed we had provided 15 areas that had been identified by our assisted dying research network as possibly being important (screenshare list of 15). These are in order based on the 119 completed survey respondents’ answers. Did any of these areas stand out for you as particularly important? Unimportant? Is anything missing from this list?

1. What areas of assisted dying research would be most beneficial to your work/your organisation and why?
2. What role, if any, do you see yourself/organisation playing in assisted dying research?
3. How is it best to disseminate AD research to make it available and useful for clinicians and organisations?
4. What form should the outputs take?

**What’s happening in practice**

1. Assisted dying has been legally available for eight months now. Part of our research is understanding how assisted dying is changing clinical practice. At the moment, what would you say is the relationship between you/your organisation and the implementation of Assisted Dying? Can you see this changing over time? In what ways?
2. How has the legalisation of assisted dying changed you/your organisation and the work that you do?
3. From your perspective, what is working best with the End of Life Choice Act/AD service and what are the most significant barriers/challenges with it, for your work?
4. How did you/your organisation plan to support patients who might be interested in or choose assisted dying?
5. Do you have any other comments you’d like to make?

**Closing the interviews**

- That’s all the questions we have, I’m very grateful for your time and for sharing your thoughts today.
- Is there anything that we have not discussed that you feel is relevant?
- What happens next is we get this interview transcribed and we check they’ve done that accurately. Are you interested in reading your transcript; you will have an opportunity to change or add something? You can let me know if you have any other thoughts by email at any stage too within the next month.
- What’s the best address to send a voucher to you at? CD or NW?
- Thank you for sharing your views with me today. The information you have provided is very valuable and will help us to improve the AD system and pathway for people. How would you like to close your interview today? [offer to close with karakia and waiata if they hand it over to me to close]
- **Unuhia, unuhia
  Unuhia ki te uru tapu nui
  Kia wātea, kia māmā, te ngākau, te tinana, te wairua i te ara takatā
  Koia rā e Rongo, whakairia ake ki runga
  Kia tina! TINA! Hui e! TĀIKI E!**

**Prompts**

- Why’s that? How? Can you tell me more about that? Tell me what you were thinking? What do you mean by?
